# Unilateral Diaphragmatic Paralysis Presumed Secondary to Parsonage-Turner Syndrome Presenting as Orthopnea

**DOI:** 10.7759/cureus.110618

**Published:** 2026-06-10

**Authors:** Tharmesh P Sivananthan, Umair Falak, Ahmad Asim

**Affiliations:** 1 Respiratory Medicine, Gateshead Health NHS Foundation Trust, Gateshead, GBR

**Keywords:** brachial neuritis, diaphragmatic paralysis, dyspnea, neuralgic amyotrophy, orthopnea, parsonage-turner syndrome, phrenic nerve palsy, positional spirometry

## Abstract

Unilateral diaphragmatic paralysis is an uncommon cause of dyspnea and orthopnea and may be overlooked when symptoms are attributed to more common cardiopulmonary conditions. This is particularly relevant in patients with underlying chronic obstructive pulmonary disease, where progressive breathlessness may initially be attributed to worsening obstructive lung disease. Parsonage-Turner syndrome, also known as neuralgic amyotrophy, is a rare neuropathic disorder that typically presents with acute shoulder or upper limb pain followed by weakness. Phrenic nerve involvement is uncommon but can result in diaphragmatic dysfunction and positional breathlessness. We present the case of a 56-year-old woman with a background of chronic obstructive pulmonary disease who developed progressive dyspnea and orthopnea over several months. She had initially been treated for worsening chronic obstructive pulmonary disease and was later referred for cardiac assessment, which did not identify a cardiac cause. Chest imaging demonstrated elevation of the right hemidiaphragm, prompting respiratory review. Retrospective history-taking revealed that her symptoms followed a flu-like illness associated with right arm and lower chest pain, reduced grip strength, and difficulty opening lids. Examination demonstrated weakness of right thumb abduction and adduction. Diaphragm ultrasound was reported as showing complete paralysis of the right hemidiaphragm, and further cross-sectional imaging excluded alternative structural causes. Positional spirometry demonstrated a marked reduction in lung volumes when supine, supporting diaphragmatic dysfunction. Electromyography and nerve conduction studies were performed late, approximately 10 months after symptom onset, and were non-diagnostic. A clinically supported diagnosis of unilateral diaphragmatic paralysis presumed secondary to Parsonage-Turner syndrome with phrenic nerve involvement was made. Management focused on diaphragmatic physiotherapy, breathing exercises, and optimisation of chronic obstructive pulmonary disease treatment, with subsequent symptomatic and spirometric improvement. This case highlights the importance of considering diaphragmatic paralysis in patients with persistent unexplained orthopnea, particularly when cardiac investigations are non-diagnostic and neurological symptoms are present.

## Introduction

Orthopnea is a common symptom encountered in respiratory and acute medical practice and is most frequently associated with cardiac failure or chronic respiratory disease. However, less common neuromuscular causes of positional breathlessness may be overlooked, particularly when patients have a pre-existing cardiopulmonary comorbidity. Diaphragmatic dysfunction is an uncommon and under-recognised cause of dyspnea and can range from asymptomatic radiographic elevation of a hemidiaphragm to exertional dyspnea, orthopnea, sleep-disordered breathing, and respiratory failure, depending on severity and whether involvement is unilateral or bilateral [1,2]. In patients with chronic obstructive pulmonary disease, diaphragmatic dysfunction may mimic worsening obstructive lung disease, contributing to diagnostic delay.

Unilateral diaphragmatic paralysis has a broad differential diagnosis, including trauma, thoracic surgery, malignancy, infection, cervical spine pathology, subphrenic pathology, diaphragmatic eventration, and neurological disorders affecting the phrenic nerve [1,2]. Diagnosis can be challenging because symptoms are often non-specific and may mimic more common cardiac or pulmonary disease. Chest radiography may show hemidiaphragm elevation, but this finding is not diagnostic in isolation. Further assessment with diaphragm ultrasound, cross-sectional imaging, and pulmonary function testing in upright and supine positions is often required to support the diagnosis and exclude alternative causes [2,3].

Parsonage-Turner syndrome, also known as neuralgic amyotrophy or brachial neuritis, is an uncommon peripheral neuropathy classically characterised by acute shoulder or upper limb pain followed by weakness and functional impairment [4,5]. A preceding viral illness may act as a trigger in some cases and can provide an important diagnostic clue when later neurological symptoms emerge [4,5]. Although Parsonage-Turner syndrome predominantly affects the brachial plexus, phrenic nerve involvement has been described and may result in diaphragmatic paralysis with orthopnea or unexplained dyspnea [6,7]. Phrenic nerve involvement is uncommon and has been reported in approximately 7.6% of patients with neuralgic amyotrophy in one cohort [6]. We present a case of unilateral diaphragmatic paralysis presumed secondary to Parsonage-Turner syndrome in a patient initially investigated for more common cardiopulmonary causes of orthopnea. This case highlights that persistent orthopnea with non-diagnostic cardiac assessment should prompt consideration of diaphragmatic dysfunction and neuromuscular causes, particularly when neurological symptoms are present.

## Case presentation

A 56-year-old woman with a background of chronic obstructive pulmonary disease was evaluated in the respiratory clinic with progressive dyspnea and orthopnea over several months. Her symptoms had initially been managed in primary care as worsening chronic obstructive pulmonary disease. However, the prominent positional nature of her breathlessness later prompted reconsideration of chronic obstructive pulmonary disease as the sole explanation for her presentation. Due to persistent orthopnea, she was referred for cardiology assessment. Echocardiography did not identify a cardiac cause for her symptoms, and the available cardiology assessment did not suggest heart failure as the primary explanation. B-type natriuretic peptide (BNP) testing and formal ischaemia evaluation were not documented in the records available for this report.

Repeat chest radiography demonstrated elevation of the right hemidiaphragm, prompting referral to respiratory medicine. At this stage, the differential diagnosis for an elevated hemidiaphragm included phrenic nerve palsy, diaphragmatic eventration, malignancy, cervical or thoracic pathology, and subphrenic disease. Subsequent CT thorax confirmed marked elevation of the right hemidiaphragm, supporting suspicion of unilateral diaphragmatic paralysis (Figure 1).

**Figure 1:**
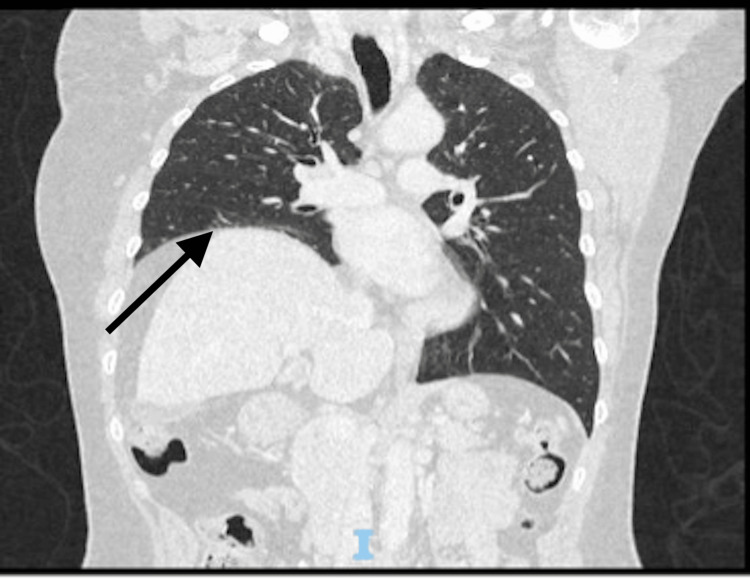
CT Thorax Demonstrating Right Hemidiaphragm Elevation Coronal CT thorax demonstrating elevation of the right hemidiaphragm. The black arrow indicates the elevated right hemidiaphragm, supporting suspicion of unilateral diaphragmatic paralysis.

On retrospective history-taking during respiratory review, she described a distinct sudden worsening of symptoms several months earlier, beginning with a flu-like illness associated with pain in the right arm and lower chest. She also reported difficulty gripping objects and weakness in her right hand, particularly when opening jar lids. Neurological examination documented weakness of right thumb abduction and adduction, consistent with the patient’s reported reduced grip strength and difficulty opening lids. Detailed Medical Research Council (MRC) grading, sensory examination findings, and reflex assessment were not available in the documentation reviewed.

Diaphragm ultrasound was reported as showing complete paralysis of the right hemidiaphragm; however, detailed objective ultrasound parameters, including diaphragmatic excursion, paradoxical motion, or sniff manoeuvre findings, were not available in the records reviewed. CT imaging of the thorax, abdomen, and pelvis, along with MRI imaging of the head and cervical spine, did not identify an alternative structural, malignant, or neurological cause. Spirometry was performed in both standing and supine positions and demonstrated a significant reduction in lung volumes when supine, with forced expiratory volume in one second falling from 1.71 L to 0.90 L and forced vital capacity falling from 2.22 L to 1.28 L. A fall in forced vital capacity of more than 20-30% from upright to supine values is suggestive of diaphragmatic weakness, and these findings supported clinically significant diaphragmatic dysfunction. The patient’s baseline and follow-up spirometry results are summarised in Table 1.

**Table 1:** Standing and Supine Spirometry Findings Supine spirometry demonstrated a marked reduction in FEV1 and FVC compared with standing values, supporting clinically significant diaphragmatic dysfunction. Follow-up standing spirometry showed improvement after physiotherapy and optimisation of chronic obstructive pulmonary disease management. FEV1: forced expiratory volume in one second; FVC: forced vital capacity.

SPIROMETRY PARAMETER	INITIAL STANDING	INITIAL SUPINE	FOLLOW-UP STANDING
FEV1, L (% PREDICTED)	1.71 (72%)	0.90 (38%)	1.98 (91%)
FVC, L (% PREDICTED)	2.22 (75%)	1.28 (43%)	2.66 (99%)
FEV1/FVC	0.77 (77%)	0.70 (70%)	0.74 (74%)

The combination of preceding viral illness, right arm pain, hand weakness, elevated right hemidiaphragm, ultrasound-reported right hemidiaphragm paralysis, marked reduction in supine spirometry, and exclusion of alternative structural causes led to a clinically supported diagnosis of unilateral diaphragmatic paralysis presumed secondary to Parsonage-Turner syndrome with phrenic nerve involvement. Electromyography and nerve conduction studies were performed late, approximately 10 months from symptom onset, and did not demonstrate active neuritis. Given the delayed timing of testing, these findings were not considered sufficient to exclude Parsonage-Turner syndrome, as electrophysiological abnormalities may be less evident once the acute phase has passed.

As the patient presented late in the disease course, corticosteroid therapy was not initiated. She was referred to physiotherapy for diaphragmatic breathing exercises, and her chronic obstructive pulmonary disease management was optimised. Follow-up spirometry after three months demonstrated improvement in standing lung volumes, with forced expiratory volume in one second improving to 1.98 L and forced vital capacity improving to 2.66 L. Clinically, she reported symptomatic improvement following rehabilitation.

## Discussion

Diaphragmatic dysfunction is an important but often under-recognised cause of breathlessness. Clinical manifestations vary depending on whether dysfunction is unilateral or bilateral, the severity of weakness, and the presence of underlying cardiopulmonary disease. Unilateral diaphragmatic paralysis may be asymptomatic, but symptomatic patients can present with exertional dyspnea, orthopnea, sleep disturbance, or reduced exercise tolerance [1,2]. In patients with pre-existing chronic respiratory disease, these symptoms may be incorrectly attributed to progression of the underlying lung condition, contributing to diagnostic delay. In this case, the patient’s background of chronic obstructive pulmonary disease created an additional diagnostic confounder, as progressive dyspnea and reduced exercise tolerance could reasonably be attributed to worsening obstructive lung disease. However, the prominent positional nature of her symptoms and the elevated right hemidiaphragm prompted reconsideration of the diagnosis.

The differential diagnosis for unilateral diaphragmatic paralysis is broad and includes thoracic surgery, trauma, malignancy, cervical spine disease, infection, diaphragmatic eventration, subphrenic pathology, and phrenic neuropathy [1,2]. In this case, the initial clinical pathway focused on common cardiopulmonary causes of orthopnea, particularly worsening chronic obstructive pulmonary disease and cardiac failure. This was reasonable given the presenting symptom profile, but cardiology assessment and echocardiography did not identify a cardiac cause for her symptoms, and persistent positional breathlessness prompted reconsideration of the diagnosis. The elevated right hemidiaphragm on chest imaging was an important radiological clue that shifted the diagnostic focus towards diaphragmatic dysfunction, although this finding was not diagnostic in isolation. Diaphragm ultrasound, cross-sectional imaging, MRI, and positional spirometry were therefore used to support diaphragmatic paralysis and exclude alternative structural causes.

Parsonage-Turner syndrome, also known as neuralgic amyotrophy or brachial neuritis, is an uncommon peripheral neuropathy characterised by acute pain followed by weakness, classically affecting the shoulder girdle and upper limb [4,5]. A preceding infective illness has been reported in a proportion of patients, supporting an immune-mediated mechanism in some cases [4,5]. Although the brachial plexus is most commonly affected, extra-brachial involvement can occur. Phrenic nerve involvement is a recognised but uncommon manifestation, reported in approximately 7.6% of patients with neuralgic amyotrophy in one cohort, and may lead to unilateral or bilateral diaphragmatic paralysis [6,8]. Previous reports have highlighted that phrenic nerve involvement may present predominantly with dyspnea or orthopnea, leading to diagnostic delay when more common cardiopulmonary causes are considered first [7,9,10]. In this patient, retrospective history-taking revealed a preceding flu-like illness, right arm pain, reduced grip strength, difficulty opening lids, and thumb weakness. These features provided important clinical clues supporting a presumed diagnosis of Parsonage-Turner syndrome with phrenic nerve involvement.

This case reinforces the importance of detailed and repeated history-taking in patients with unexplained breathlessness. Orthopnea often triggers investigation for cardiac failure, but persistent symptoms despite non-diagnostic cardiac assessment should prompt clinicians to broaden the differential diagnosis. Positional spirometry can provide supportive physiological evidence of diaphragmatic dysfunction. A fall in forced vital capacity of more than 20-30% from upright to supine values is suggestive of diaphragmatic weakness, and the marked reduction in supine forced expiratory volume in one second and forced vital capacity in this patient supported clinically significant diaphragmatic dysfunction [2]. Diaphragm ultrasound is also a useful non-invasive investigation for assessing diaphragmatic movement [3]. Cross-sectional imaging remains important to exclude structural causes such as malignancy, compressive lesions, thoracic pathology, cervical pathology, or subphrenic disease.

The non-diagnostic electrophysiology represents an important limitation. Electromyography and nerve conduction studies were performed approximately 10 months after symptom onset, which may have reduced sensitivity for detecting active neuritis or denervation. Therefore, the presumed diagnosis was based on the overall clinical pattern, including preceding viral illness, acute pain followed by weakness, suspected phrenic nerve involvement, ultrasound-reported right diaphragmatic paralysis, positional spirometry supporting diaphragmatic dysfunction, and exclusion of alternative structural causes.

There are further limitations to this case report. Complete baseline chronic obstructive pulmonary disease staging, smoking history, mMRC score, and pre-morbid spirometry were not available in the documentation reviewed. This limits direct comparison between her baseline obstructive lung disease and subsequent diaphragmatic dysfunction. In addition, although cardiology assessment and echocardiography did not identify a cardiac cause for her orthopnea, BNP testing and formal ischaemia assessment were not available in the documentation reviewed. The neurological examination was also limited by the retrospective nature of the case report, as detailed MRC grading, reflexes, and sensory findings were not available. Finally, the diaphragm ultrasound report documented complete right hemidiaphragm paralysis, but detailed ultrasound parameters such as excursion measurements, paradoxical movement, and sniff manoeuvre findings were not available for inclusion.

Management of Parsonage-Turner syndrome depends on timing and severity. Early treatment is often focused on analgesia, maintaining function, and in selected cases, corticosteroid therapy, although evidence for steroid benefit remains uncertain [5]. In delayed presentations, as in this case, rehabilitation and physiotherapy are central to management. Diaphragmatic breathing exercises, optimisation of underlying respiratory disease, and functional rehabilitation may support symptomatic improvement. The subsequent improvement in spirometry in this patient supports the value of conservative management and respiratory physiotherapy in selected cases.

The key learning point from this case is that orthopnea is not synonymous with cardiac failure or progression of chronic obstructive pulmonary disease. In patients with persistent positional breathlessness, non-diagnostic cardiac assessment, and radiological hemidiaphragm elevation, diaphragmatic paralysis should be actively considered [1,2,9,10]. Associated neurological symptoms, even if subtle or identified retrospectively, may provide the clue to an underlying diagnosis such as Parsonage-Turner syndrome with phrenic nerve involvement.

## Conclusions

Parsonage-Turner syndrome should be considered as an uncommon but important differential diagnosis in patients presenting with unexplained orthopnea, particularly when cardiac investigations are non-diagnostic and symptoms are associated with a preceding viral illness, upper limb pain, or neurological weakness. This case highlights the importance of considering diaphragmatic paralysis in patients with persistent positional breathlessness and radiological hemidiaphragm elevation, especially when symptoms may otherwise be attributed to cardiac disease or progression of chronic obstructive pulmonary disease. Careful and repeated history-taking, diaphragm imaging, positional spirometry, and exclusion of structural causes are key to supporting the diagnosis. Early recognition may reduce diagnostic delay and allow appropriate rehabilitation, respiratory optimisation, and follow-up.
